# Scale dependency of conservation outcomes in a forest‐offsetting scheme

**DOI:** 10.1111/cobi.13362

**Published:** 2019-07-30

**Authors:** Marta Lisli Giannichi, Yoni Gavish, Timothy R. Baker, Martin Dallimer, Guy Ziv

**Affiliations:** ^1^ School of Geography University of Leeds Garstang North Leeds LS2 9JT U.K.; ^2^ Sustainability Research Institute, School of Earth and Environment, Maths/Earth and Environment Building University of Leeds Leeds LS2 9JT U.K.

**Keywords:** Amazon, avoided deforestation, conservation, offsets, private lands, restoration, spatial scale, Amazonía, compensaciones, conservación, deforestación evitada, escala espacial, restauración, tierras privadas, 亚马逊, 避免森林采伐, 保护, 补偿, 私有土地, 恢复, 空间尺度

## Abstract

Offset schemes help avoid or revert habitat loss through protection of existing habitat (avoided deforestation), through the restoration of degraded areas (natural regrowth), or both. The spatial scale of an offset scheme may influence which of these 2 outcomes is favored and is an important aspect of the scheme's design. However, how spatial scale influences the trade‐offs between the preservation of existing habitat and restoration of degraded areas is poorly understood. We used the largest forest offset scheme in the world, which is part of the Brazilian Forest Code, to explore how implementation at different spatial scales may affect the outcome in terms of the area of avoided deforestation and area of regrowth. We employed a numerical simulation of trade between buyers (i.e., those who need to offset past deforestation) and sellers (i.e., landowners with exceeding native vegetation) in the Brazilian Amazon to estimate potential avoided deforestation and regrowth at different spatial scales of implementation. Allowing offsets over large spatial scales led to an area of avoided deforestation 12 times greater than regrowth, whereas restricting offsets to small spatial scales led to an area of regrowth twice as large as avoided deforestation. The greatest total area (avoided deforestation and regrowth combined) was conserved when the spatial scale of the scheme was small, especially in locations that were highly deforested. To maximize conservation gains from avoided deforestation and regrowth, the design of the Brazilian forest‐offset scheme should focus on restricting the spatial scale in which offsets occur. Such a strategy could help ensure conservation benefits are localized and promote the recovery of degraded areas in the most threatened forest landscapes.

## Abstract 2

*Article impact statement*: Conservation offset schemes may yield greater additionality through avoided deforestation and restoration if implemented in smaller areas.

## Resumen

Los esquemas de compensación ayudan a evitar o revertir la pérdida de hábitat mediante la protección del hábitat existente (deforestación evitada), mediante la restauración de áreas degradadas (recrecimiento natural) o ambos. La escala espacial de una mitigación puede influir en cuál de ellos es seleccionado y es un aspecto importante del diseño de esquema. Sin embargo, no se entiende bien cómo influye la escala espacial en las compensaciones entre la preservación del hábitat existente y la restauración de áreas degradadas. Utilizamos el esquema de compensación forestal más grande del mundo, que forma parte del Código Forestal Brasileño, para explorar cómo la implementación a diferentes escalas espaciales puede afectar el resultado en términos de la superficie de deforestación evitada y el área de recrecimiento. Empleamos una simulación numérica del comercio entre compradores (i. e., aquellos que necesitan compensar la deforestación pasada) y vendedores (i. e., propietarios con exceso de vegetación nativa) en la Amazonía brasileña para estimar deforestación evitada y el recrecimiento a diferentes escalas espaciales de implementación. Permitir compensaciones en grandes escalas espaciales dio lugar a una superficie de deforestación evitada 12 veces mayor que de recrecimiento, mientras que restringir compensaciones a pequeñas escalas espaciales dio lugar a una superficie de recrecimiento dos veces mayor que la deforestación evitada. La mayor superficie total (deforestación evitada y recrecimiento combinados) se conservó cuando la escala espacial del esquema era pequeña, especialmente en localidades muy deforestadas. Para maximizar los beneficios de conservación derivados de la deforestación evitada y el recrecimiento, el diseño del esquema brasileño de compensaciones debe centrarse en restringir la escala espacial en la que se producen las compensaciones. Esta estrategia ayudaría a garantizar que los beneficios de la conservación sean localizados y promuevan la recuperación de zonas degradadas en los paisajes forestales más amenazados.

## 摘要

生态补偿计划通过保护现有生境 (避免森林砍伐) 、恢复退化生境 (自然再生) 或双管齐下的方式, 来帮助避免和逆转生境丧失。补偿计划实施的空间尺度可能会影响以上两个方面的侧重性, 因此, 其在补偿计划的设计中十分重要。然而, 有关空间尺度如何影响现有生境保护和退化生境恢复之间的利弊权衡却研究甚少。本研究中, 我们用世界上最大的森林补偿计划 (来自《巴西森林法典》) 来探索在不同尺度下实施补偿计划对于森林免遭砍伐的面积和森林再生面积的保护成效的影响。我们在巴西亚马逊地区采用买家 (即需要补偿已经砍伐的森林的人) 和卖家 (即拥有大量原生植被的土地所有者) 之间进行贸易的数值模拟方法, 评估了不同空间尺度下实施补偿计划可能带来的森林免伐和森林再生的成效。结果显示, 如果允许在较大空间尺度上进行补偿, 则森林免伐面积可能比森林再生面积大十二倍;而在小尺度上进行补偿, 可能导致森林再生面积比避免砍伐的面积大一倍。当补偿计划的空间尺度很小, 特别是实施地点在森林砍伐十分严重的地区时, 则得到保护的森林总面积 (森林免伐和森林再生面积之和) 最大。为了从森林免伐和森林再生中获得最大保护收益, 巴西森林补偿计划的设计应该着重于限制进行补偿的空间尺度。这一策略有助于保障当地的保护效益, 并促进受到严重威胁的森林景观中的退化生境的恢复。 **【翻译: 胡怡思; 审校: 聂永刚】**

## Introduction

A variety of mechanisms have been developed to manage human‐caused habitat change and promote outcomes that aid conservation (Betts et al. 2017). Some of these systems incentivize landowners to follow good environmental practices (e.g., subsidy payments and payments for ecosystem services) (Pirard 2012), whereas others legislate to ensure past or future environmental disturbances are compensated for (e.g., tradable permits or habitat and biodiversity offset schemes) (Santos et al. 2014). The latter group operates as markets, in which environmental goods are traded between landowners who supply the market goods (i.e., sellers) and those who need to compensate for environmental damage (i.e., buyers) (Ring et al. 2010).

Offset schemes have gained popularity around the globe due to the straightforward logic of trading environmental losses for equivalent conservation gains, although there has been concern whether such equivalency can, in fact, be achieved (Bull et al. 2013, 2015). To compensate for environmental loss, offset schemes typically use averted loss, restoration, or both as offset strategies (Maron et al. 2012). Averted loss targets the protection of existing biodiversity and natural habitat potentially at risk of being lost, whereas restoration favors the recovery of degraded habitats and promotes secondary vegetation (Curran et al. 2014; Maron et al. 2016).

The trade‐offs between the advantages and disadvantages of conservation schemes that favor either averted loss or restoration have been extensively debated (e.g., Maron et al. 2012; Gardner et al. 2013; Curran et al. 2014; Quétier et al. 2015). Although the length of time restoration requires increases the risk of failure (Drechsler & Hartig 2011; Maron et al. 2012), this strategy may be attractive if restoration occurs on site or near affected areas (Wissel & Wätzold 2010). For example, where negative impacts are caused by land‐cover change to pasture or agriculture, such as in many tropical forest regions, restoration via natural regrowth has been promoted to recover degraded land and enhance secondary forest cover (Chazdon et al. 2016; Strassburg et al. 2016). In contrast, averted loss can protect old‐growth vegetation, but to result in effective conservation gains, the area protected needs to be ecologically equivalent (i.e., the same vegetation type) as the damaged site (Bull et al. 2013). The protection also needs to occur in sites where threats or development pressure are imminent so as to generate benefits that would not occur in the absence of the scheme—a concept called “additionality” (Maron et al. 2010).

A key element that determines the effectiveness of averted loss and restoration is the spatial location of the offset (Gonçalves et al. 2015). Studies that use conservation‐planning approaches to identify the spatial scale (e.g., local or regional) where potential offsets should be located typically consider specific biological targets or habitat characteristics (e.g., species distributions or the presence of certain taxa) to determine where offsets should occur (e.g., Kiesecker et al. 2009; Gordon et al. 2011; Kujala et al. 2015). These studies indicate both local (e.g., site and microwatershed) and regional (e.g., biome and river basin) spatial scales have the potential to achieve averted loss and restoration goals if offsets are placed in strategically defined areas. Conservation‐planning approaches have been particularly useful in offset schemes that explicitly include biodiversity metrics in their offset strategies (Gordon et al. 2011). However, some offset schemes have simpler offset conditions (e.g., a hectare of loss for a hectare of gain) that do not include specific biodiversity metrics (McKenney & Kiesecker 2010). In these cases, conservation‐planning approaches cannot be used so readily to determine the optimal location of offsets. For these schemes, administrative boundaries, such as the limits to municipalities, states, or counties, may be an appropriate way to influence the spatial scale and location of offsets to facilitate environmental governance and potentially maximize conservation benefits (Nepstad et al. 2013; Boyd et al. 2018).

Administrative boundaries have been used to define the spatial scales of offset schemes in the United States (e.g., for conservation banking and transferable development rights, McKenney & Kiesecker 2010) and in Brazil (Brazilian Forest offsets, Soares‐Filho et al. 2014). They represent well‐known jurisdictions in which many policy decisions already operate and therefore facilitate the implementation of offset markets. Some studies suggest that averted loss may not be achieved within offset schemes that use small administrative boundaries to limit trade because this restriction will lead to a reduced number of sellers and little area available for compensation (Chomitz 2004; McConnell & Walls 2009). Conversely, in some offset schemes, the use of larger administrative boundaries to expand trade may lead to limited additionality. In these cases, areas not under development pressure are likely to absorb the offsets that the scheme requires because these areas will tend to have low opportunity costs and outcompete areas under deforestation pressure that are typically associated with high opportunity costs (Santos et al. 2014). Hence, only areas that would likely remain untouched even in the absence of the scheme may ultimately be protected, and scheme additionality will be very low (McConnell & Walls 2009; Freitas et al. 2017). However, explicit tests of how the spatial scale of offsets may alter the trade‐offs between averted loss and restoration, and overall scheme additionality, have not been performed.

We quantified the effect of scale on the trade‐offs between averted loss and restoration as additional conservation outcomes of an offset scheme included in the Brazilian Forest Code (FC) (officially known as the Brazilian Native Vegetation Protection Law [Brasil 2012]). The FC regulates the protection of native vegetation on private land (Azevedo et al. 2017) and requires that a certain percentage of the property be set aside and managed for conservative purposes, referred to as legal reserves (LRs) (Freitas et al. 2017). The percentages vary according to the biome, ranging from 20% (in the Atlantic Forest) to 80% (in the Amazon). Landowners who have LRs below these limits (i.e., is a buyer) due to past deforestation must offset their LR deficit. Buyers can offset by acquiring private land inside protected areas and PAs that are pending regularization, leasing existing native vegetation of landowners who have LRs that exceed the limits (i.e., sellers) and can be legally deforested (e.g., >80% in the Amazon), or allowing natural forest regrowth in their LR deficit (see details in Methods).

We focused on the Amazon, the world's largest standing forest, which covers 400 million hectares (Assunção et al. 2017) and holds nearly 26% of total carbon stored in tropical forests (Baccini et al. 2012). Since 2014, deforestation rates in the Brazilian Amazon have risen and endanger national commitments to reduce carbon emissions from deforestation (Rochedo et al. 2018). We used avoided deforestation to represent averted loss and natural regrowth as a restoration strategy because these are the principal conservation outcomes of the offset strategies within this example. We employed different administrative boundaries as approximations of different spatial scales (i.e., small to large) and compared the effect of scale on conservation outcomes across a range of policy scenarios. The administrative boundaries represent regions over which the scheme could be implemented and are well‐established jurisdictions. We hypothesized that allowing offsets across large spatial scales yields more avoided deforestation than regrowth (but not necessarily more additionality), given the number of sellers available to offset, whereas the opposite occurs at small spatial scales. We expected intermediary spatial scales to yield similar gains from avoided deforestation and regrowth. We estimated regrowth and avoided deforestation using numerical simulation of offset trade between >370000 buyers and sellers and considered our results in light of the current implementation guidelines for the FC and other offsetting policies.

## Methods

### Details of Case Study

In Brazil, private lands within established PAs need to be expropriated by the statutory environmental agency because they are still under private ownership. The FC states that such process can be used as an offset option by buyers. In this case, buyers must purchase an entire property (including cleared portions) inside a PA that is at least equivalent to their LR deficit and donate to the environmental agency (option 1). This option allows for a perpetual solution for buyers, which appears to be their preferred option (Giannichi et al. 2018).

The lease of existing native vegetation is called Environmental Reserve Quota (CRA) (Cota de Reserva Ambiental). The CRA is a hectare‐by‐hectare market, wherein a buyer can lease the extra LRs of several sellers or one seller can supply several buyers (option 2). Instead of a single perpetual transaction as the acquisition of land in PAs, CRA consists of contracts in which the price of the hectare and the duration of the contract are decided between buyers and sellers. Some buyers may prefer to offset their LR deficit by allowing the natural regrowth of secondary forest (option 3) (Soares‐Filho et al. 2016).

We used a land‐tenure database (Freitas et al. 2017) to acquire landowners’ property boundaries and land‐cover data sets (TerraClass, De Almeida et al. [2016], and Global Forest Change, Hansen et al. [2013]) to calculate the extent of the LR at property level. Based on the LR extent, we classified them into buyers or sellers. According to the FC, a landowner is a potential buyer if LR deforestation occurred prior to 2008. If the LR currently exceeds 80%, the property was classified as a seller. On private lands, LR that exceeds 80% can be legally deforested, up to that same limit. Although native vegetation below this amount cannot be deforested (so its protection in an offset scheme is not additional), in some cases landowners who have LR below 80% are also eligible to supply the market. These cases include smallholders (i.e., family‐managed properties <400 ha) and settlements (i.e., former megaproperties that were underused and allotted and distributed to families) that can offer any amount of LR within their property. Private properties inside PAs were also classified as sellers, and their native vegetation was considered nonadditional because its standing native vegetation is already protected and cannot be legally deforested. Supporting Information contains details on the FC, the data sets used, and the classification of buyers and sellers.

### Offset Spatial Scales and Policy Scenarios

**Figure 1:**
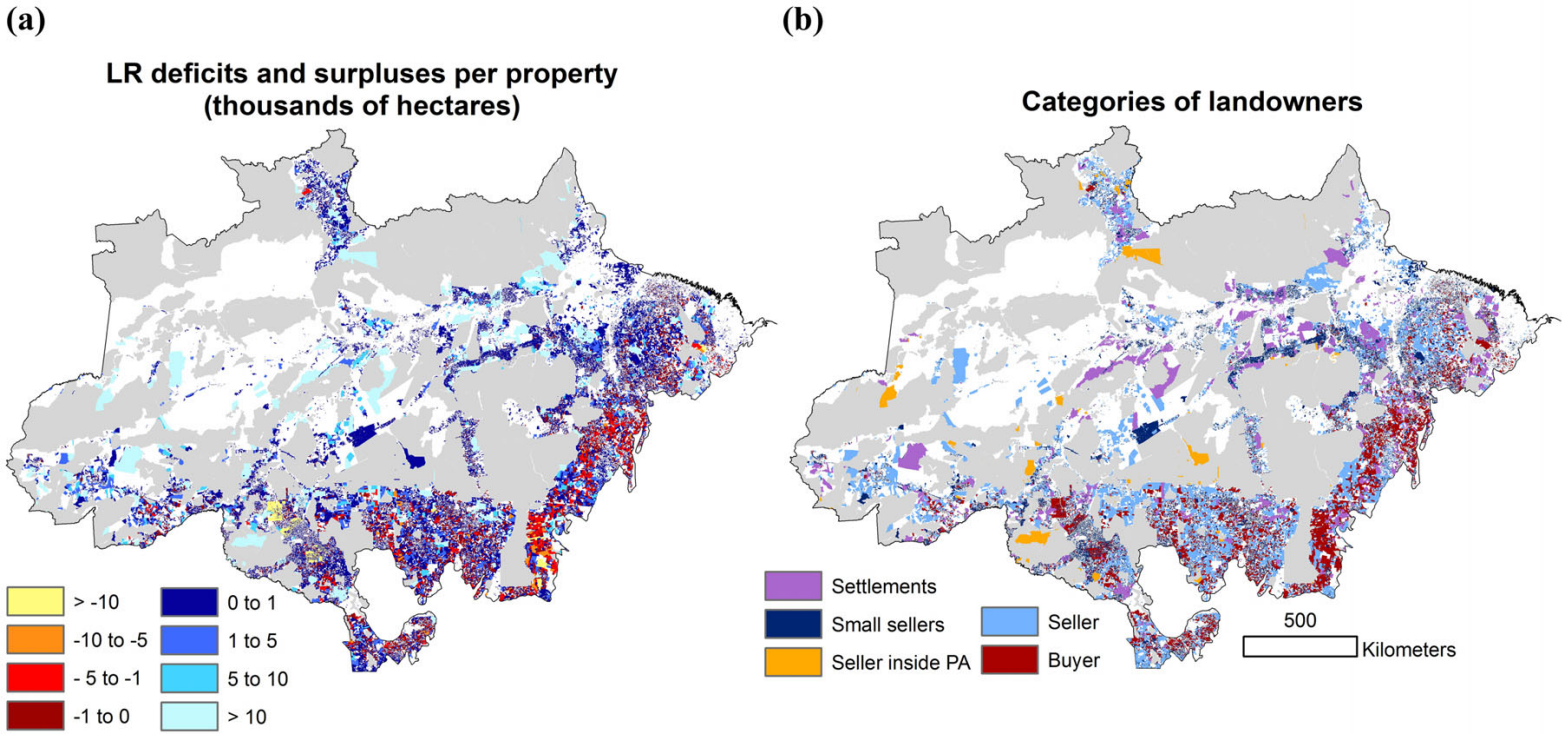
Across the Amazon biome, the (a) distribution and size of legal reserve (LR) deficits of buyers (i.e., native vegetation below the limits established by law, for the Amazon biome <80%) and surpluses of sellers at the property level (reds, deficits; blues, surpluses) and (b) categories of landowners: buyers (i.e., landowners who have LR deficits), sellers (i.e., landowners with LR surpluses), settlements, small sellers, and sellers inside protected areas (white, unassessed areas, such as areas with no land titles gray, protected areas).

We considered 5 different nested administrative boundaries as offset spatial scales (Table 1 & Fig. 1), from the large (biome) to small (municipality). The FC states that offsets must occur within the same biome. If between states, offsets must occur in areas identified as priorities for conservation (Supporting Information). The FC does not account for ecological equivalence between buyers and sellers to offset forest loss (e.g., biomass or biodiversity equivalence). Besides the boundaries mentioned in the law (biome and state), we used other 3 nationally established administrative boundaries (Table 1) that could facilitate implementation. The FC offset scheme has not been fully regulated; thus, offset scales can still be amended once each state legislates their own offsetting rules. Apart from biome, all administrative boundaries include several individual polygons that vary in size (Table 1).

**Table 1:** Number of polygons that comprises each spatial scale (administrative boundary) and respective deciles of polygon sizes in the Brazilian Amazon biome

	Polygons	1st decile (Mha)	5th decile (Mha)	9th decile (Mha)
Biome*	1	–	–	–
State	9	9.82	22.43	131.00
Mesoregion	26	1.65	9.26	38.67
Microregion	81	0.55	3.11	12.14
Municipality	499	0.04	0.30	2.12

^*^A single unit of 422 million ha; thus, there are no deciles.

For each spatial scale, we considered the following 3 policy scenarios (Supporting Information). Offsets occurred in all PAs, CRA, and regrowth (scenario 1). Offsets were allowed on private land inside all PAs. The text in the FC is not specific about whether the PA must be a federal, state, or municipal area. Thus, this scenario included offsets in all PAs first, followed by CRA offset trade, and then the remaining buyers were assigned to offset through regrowth.

Offsets occurred in federal PAs, CRA, and regrowth (scenario 2). The Ministry of the Environment established a regulatory framework (Brasil 2016) that considers only federal PAs for compensation. As states still need to legislate their offset rules, we included this framework as a scenario and excluded offsets in state and municipal PAs first, followed by CRA offset trade, and then remaining buyers were assigned to offset through regrowth.

Offsets occurred with CRA and regrowth (scenario 3). This scenario ruled out offsets on private land inside any PA and included only CRA trade first, and then remaining buyers were assigned to offset through regrowth.

Scenario 1 included all offset options stated by law—the most permissive scenario. Scenarios 2 and 3 gradually imposed policy restrictions associated with the offsetting options. The different policy scenarios were established to examine whether the results of our simulations would be driven by the spatial scales or by the different rules of the policy scenarios. For example, offset in PAs is unique to the FC, and removing this option allowed a more general examination of the effect of scale on offset schemes outcomes.

We assumed compliance is a buyer‐led strategy because noncompliance incurs severe penalties, such as fines, land embargoes, or denial of access to loans. Therefore, buyers actively sought sellers in our analysis. Offset in PAs appears to be preferred by buyers because, besides being in perpetuity (Giannichi et al. 2018), they tend to be low cost (Freitas et al. 2017). Thus, in policy scenarios 1 and 2, we first attempted to exhaust demand inside the respective PAs.

To simulate offset (PAs and CRA), we developed an algorithm (Supporting Information) in which each buyer sought the best‐matching seller. The algorithm is deterministic, meaning the deals between buyers and sellers are established based on the smallest difference between buyer's deficit and seller's surplus. In the case of offsetting in PAs, buyers must acquire an area that is at least equivalent to their deficit; thus, only one transaction was allowed. We assumed that, given their low cost, buyers would be willing to purchase an area that was up to 20% larger than their LR deficit. If such conditions were met, a buyer was considered compliant. If not, a buyer remained noncompliant and available for CRA trade.

The CRA is a hectare‐by‐hectare offset market; thus, buyers looked for sellers that had the most similar area of native vegetation surplus to their deficit. Buyers were considered compliant if they managed to offset all their deficit in 3 transactions (3 transactions were also established for sellers). We assumed a limited number of transactions because the general behavior of landowners is to minimize transaction costs associated with each trade (Reid et al. 2015). If a buyer remained noncompliant after CRA trade, they were automatically allocated to offset through natural regrowth by default.

The assumptions above are simplifications of a complex and embryonic offset policy. There is still no data on offsets in PAs and CRA as they are still in early or pending regulatory stages. We submitted our simulations to a sensitivity analysis (Supporting Information) in which we progressively increased the 20% limit of private land inside PAs and increased the number of transactions allowed in CRA to assess whether our results are robust.

The best‐match algorithm was iterated for each of the 3 policy scenarios at each of the 5 spatial scales. After each of the 15 simulations, we computed the sum of total offset (in Mha) for each of the 3 compliance options: offset in PAs, CRA, and regrowth. Offset in PAs was divided into 2 conservation outcomes: the area of potential regrowth and the area representing nonadditional offset. Private land in PAs, after acquired by buyers, must be donated to the statutory environmental agency, making their cleared portions likely to be allocated to regrowth. The area covered by natural vegetation was therefore considered nonadditional because it is already protected. Total offset with CRA was also divided into 2 conservation outcomes: avoided deforestation and nonadditional offset. Avoided deforestation corresponded to offsets occurring in unprotected native vegetation (e.g., vegetation that can be deforested). Last, offsets through regrowth outside PAs were computed as a single conservation outcome.

To calculate total additionality for each simulation, we summed avoided deforestation and regrowth (inside and outside PAs), assuming these are both conservation benefits that would not occur in the absence of the offset scheme. Nonadditional outcomes represented offsets in already protected vegetation that are mostly in the land of smallholders and settlements. Thus, the key conservation outcomes of this analysis were avoided deforestation, regrowth, and total additionality. Finally, for each policy scenario and spatial scale, we calculated the percentages of each conservation outcome based on the total deficit to assess the proportion of total forest deficit that was effectively converted to a conservation gain.

## Results

The total native vegetation deficit across the Brazilian Amazon was 4.94 Mha, whereas the total supply of native vegetation that could be used for compensation was 10 times greater (50 Mha) (Fig. 1). Of this, 8.8 Mha could be legally deforested according to current legislation. Of the 41 Mha that could not be legally deforested, 17.8 Mha was in settlements and 13 Mha in already protected vegetation, such as private land inside PAs. Small landholdings and nonadditional sellers could offer 8.5 and 1.5 Mha, respectively. There were substantial differences in the spatial distribution of the deficit. Mato Grosso, Pará, and Rondônia contributed 80% of the total deficit; northern Mato Grosso and southeastern Pará contained around half of the deficit (2.3 Mha). These regions are inevitably likely to absorb much of the demand for surplus.

### Effect of Spatial Scale

**Figure 2:**
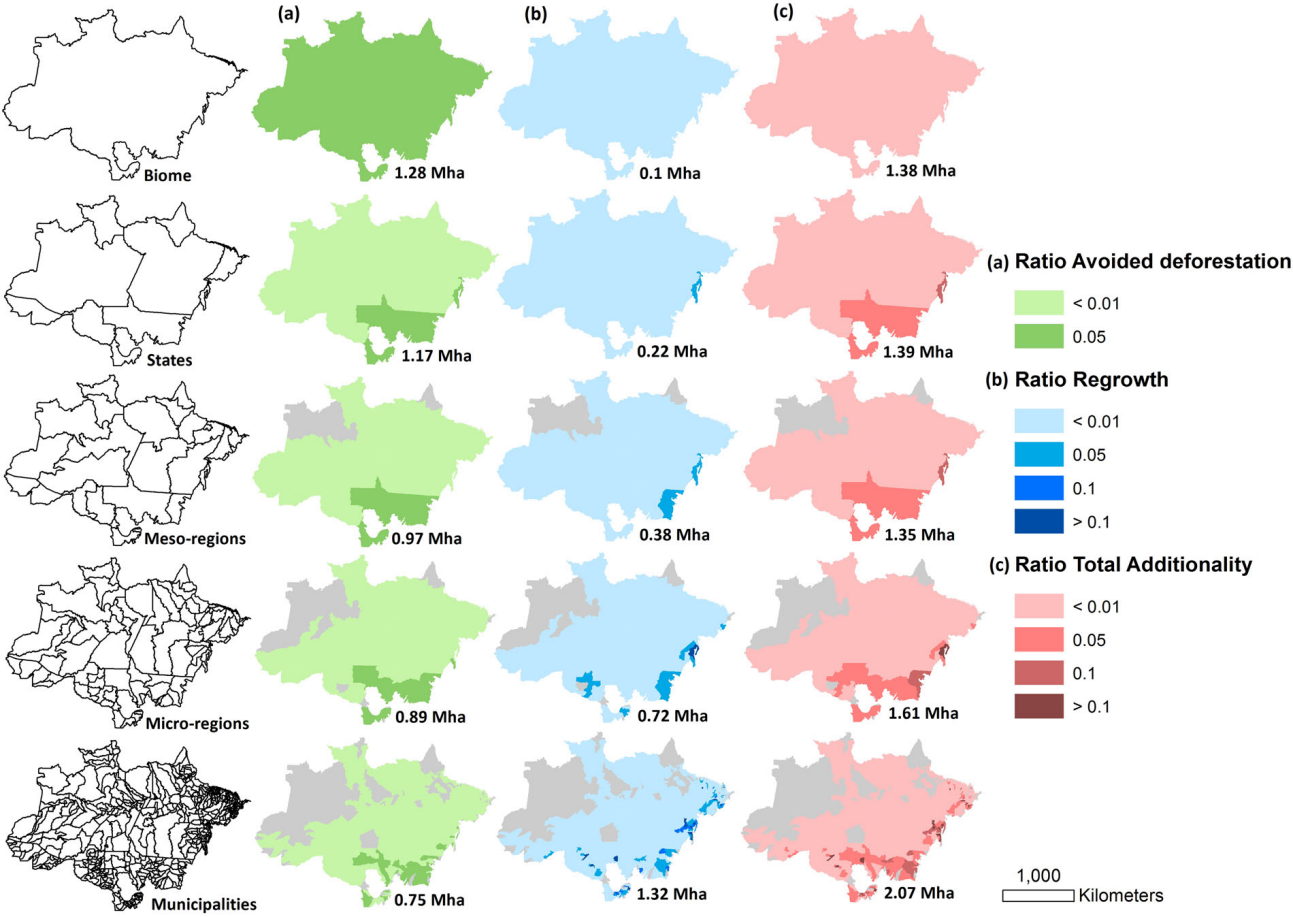
Expected spatial distribution and extent of (a) avoided deforestation, (b) regrowth, and (c) total additionality at nested spatial scales (rows: biome, state, mesoregion, microregion, and municipality [Table 1], respectively, from left to right) for policy scenario 1 (purchase land inside PAs). Maps show conservation outcomes as a proportion of the total area of the spatial scale units (the darker the shade, the greater the conservation outcome; light gray, areas with no buyers; numbers under each map, sum of the given conservation outcome across all spatial scale units).

Simulations showed 3 main results. First, as scales became smaller, the area of offsets via avoided deforestation decreased and the area of offsets via regrowth increased (Table 2 & Fig. 2). Across all scenarios, offsets via avoided deforestation remained higher than regrowth at all scales, except at the municipality level, where more offsets were allocated to regrowth. This pattern was observed because although some municipalities hold large amounts of forest deficit and little surplus, others have vast amounts of surplus and very little deficit (Fig. 1). At the municipality level, this imbalance became more evident because municipalities with large amounts of deficit had little surplus to offset. Consequently, as scale decreased, we observed an increased contribution of regrowth to total additionality and a decreased contribution of avoided deforestation (Fig. 2).

**Table 2:** Total offsets and conservation outcomes in purchasing privately owned protected areas (option 1), lease existing native vegetation through Environmental Reserve Quota (CRA trade; option 2), and allowing regrowth on the property of the buyer (option 3) for each spatial scale of the Brazilian Amazon biome (biome, state, mesoregion, microregion, and municipality) and policy scenario.^a^

		Option 1 purchase protected areas (Mha)		Option 2 lease through CRA Trade (Mha)		Option 3 regrowth in own property (Mha)		
Policy scenario	Spatial scale^b^	offset	regrowth^*^	offset	avoided^*^	offset^*^	Total additionality	Total nonadditionality
1	biome	1.36	0.1 (2)	3.58	1.28 (25.8)	0.0005 (0.01)	1.38 (27.8)	3.56 (72.2)
	state	1	0.09 (1.9)	3.82	1.17 (23.6)	0.13 (2.6)	1.39 (28.1)	3.55 (71.9)
	meso	0.68	0.07 (1.3)	3.95	0.97 (19.6)	0.31 (6.2)	1.35 (27.1)	3.59 (72.9)
	micro	0.47	0.06 (1.2)	3.81	0.89 (18.1)	0.66 (13.3)	1.61 (32.6)	3.33 (67.4)
	municipality	0.24	0.03 (0.6)	3.41	0.75 (15.1)	1.29 (26.2)	2.07 (41.9)	2.87 (58.1)
2	biome	0.36	0.005 (0.1)	4.58	1.48 (30)	0.0004 (0.0008)	1.48 (30.1)	3.46 (69.9)
	state	0.18	0.003 (0.07)	4.63	1.3 (27.5)	0.13 (2.6)	1.43 (30.7)	3.51 (69.3)
	meso	0.17	0.003 (0.07)	4.45	1.1 (22.6)	0.32 (6.5)	1.42 (29.2)	3.52 (70.8)
	micro	0.06	0.001 (0.03)	4.20	1 (21.2)	0.68 (13.8)	1.68 (35)	3.26 (65)
	municipality	0.01	0.0003 (0.007)	3.72	0.84 (17)	1.21 (24.4)	2.05 (41.4)	2.89 (58.6)
3	biome	**–**	**–**	4.94	1.64 (33.2)	0.0004 (0.0008)	1.64 (33.2)	3.3 (66.8)
	state	**–**	**–**	4.73	1.48 (29.9)	0.21 (4.3)	1.69 (34.2)	3.25 (65.8)
	meso	**–**	**–**	4.62	1.20 (24.2)	0.32 (6.5)	1.52 (30.7)	3.42 (69.3)
	micro	**–**	**–**	4.26	1.06 (21.5)	0.68 (13.8)	1.74 (35.3)	3.2 (64.7)
	municipality	**–**	**–**	3.63	0.84 (17)	1.31 (26.5)	2.15 (43.5)	2.76 (56.6)

a The sum of offsets for spatial scale and policy scenario over all options corresponds to the total native vegetation deficit (4.94 Mha). Offsets inside protected areas (PAs) result in regrowth inside PAs as a conservation outcome, whereas offsets with CRA result in avoided deforestation (avoided). Regrowth outside PAs is a single conservation outcome by itself. Total additionality is the sum of avoided deforestation, regrowth inside PA, and regrowth within a property (columns with an asterisk), and total nonadditionality is the sum of offsets in already‐protected standing vegetation. Numbers in parentheses are percentages of the areas allocated to each conservation outcome calculated based on the total deficit. The percentage of total additionality and nonadditionality sums to 100%.

b Defined in Table 1.

Second, the total area directed to conservation was larger in an offset scheme implemented at smaller scales than in a scheme allowing offsets over large scales. Using scenario 1 as an example (Fig. 2), 2.07 Mha (41.9%) of the total deficit resulted in avoided deforestation and regrowth at the municipality level, whereas at the biome level, 1.38 Mha (27.8%) of the total deficit resulted in avoided deforestation and regrowth (Table 2).

Third, spatial scales also altered the area of offsets inside PAs. As the scale of implementation was reduced, offsets inside PAs declined substantially (Table 2). The total supply of private land inside PAs would likely be enough to absorb the entire total deficit when using larger scales (e.g., biome) and result in very little additionality. However, our simulations showed that reducing the spatial scale also reduced offsets inside PAs because high supply from PAs is less available at smaller scales.

Our sensitivity analysis showed that increasing the best‐match limit of offset in PAs from 20% to 150% resulted in an increase of only 4% of the total offset, at all scales (Supporting Information). This result indicated that even if our best‐match assumptions were more flexible, our findings would likely remain the same, and that smaller scales would still result in more additionality than larger scales.

In all scenarios, across all spatial scales, most of the offset was nonadditional. In scenarios 1 and 2, offsets in PAs generated little regrowth compared to the total offset. For CRA offsets, avoided deforestation was lower than the nonadditional offsets. Overall, the total additionality was <50% in all scenarios; scenario 3 resulted in the greatest total additionality across all scales (Table 2).

## Discussion

The offset simulation exercise showed that for the FC offset scheme, larger spatial scales achieved more avoided deforestation compared with smaller spatial scales that were associated with more regrowth. However, avoided deforestation was not substantially reduced at smaller spatial scales, meaning the greatest total benefit to conservation in terms of area was achieved at the smallest scale of offset implementation. These results were consistent across all 3 policy scenarios, demonstrating that the trade‐offs linked to scale apply regardless of whether buyers were allowed to offset for their deficit by acquiring private land within PAs.

Our findings have a range of scheme‐specific policy implications. First, the FC currently states that CRA offsets must happen in the same biome and, preferably, in the same state (Soares‐Filho et al. 2014). However, CRA offsets are still pending official regulation, and each state is entitled to restrict the offset scale within their boundaries (Freitas et al. 2017). Our findings indicate that restricting offsets to the municipality level could result in greater additionality. Second, offsets inside PAs placed within the largest scale (biome) absorbed almost one‐third of the total forest deficit, resulting in very little additionality (Table 2). However, our model showed that at a small spatial scale, offsets in PAs were substantially reduced as they become less available at local levels, particularly in areas where buyers are highly concentrated (Fig. 1). Offsets in PAs have been seen as problematic given their high supply and low additionality (Soares‐Filho et al. 2016) because these areas are already protected by law (Freitas et al. 2017). At the same time, this offset option is unlikely to be ruled out by policy makers. Because PAs become scarcer at the smallest scale, offsets in PAs could also occur at this scale to promote greater additionality and result in more avoided deforestation and regrowth offsets.

Our results also suggest that the spatial scales of implementation may influence associated ecological outcomes of the scheme. For example, a large‐scale implementation could favor the protection of old‐growth forests in the Amazon through averted loss. Considering that deforestation may likely occur in the future (Soares‐Filho et al. 2006), the protection of old‐growth forests is highly important for conservation. For example, mature Amazon forests act as a carbon sink (Brienen et al. 2015) and play an important role in mitigating carbon emissions of Amazonian nations (Phillips et al. 2017). The protection of these forests also contributes to the conservation of biodiversity‐rich areas, which are key to effectively deliver ecosystem services and functions, such as seed dispersal and carbon storage (Sobral et al. 2017). However, at large scales, offsets may take place far from where deforestation occurred, undermining ecological equivalence (Wissel & Wätzold 2010). In contrast, small‐scale implementation could favor restoration through secondary forest regrowth, which may partly counterbalance biodiversity loss from deforestation (Barlow et al. 2007), increase carbon sequestration and above‐ground biomass in degraded sites, and contribute to the connectivity of fragmented landscapes (Chazdon et al. 2016). Because the offsetting rules consider only forest area, we assumed equal weights for both averted loss and restoration in our additionality metrics. However, from an ecological perspective, averted loss and restoration will likely result in distinct conservation benefits because 1 ha of preserved old‐growth forest is ecologically different from 1 ha of secondary forest (Poorter et al. 2016; Watson et al. 2018). To better understand these ecological implications, future research could include potential ecological benefits (e.g., biomass or species similarity between deficit and offsetting areas) at different implementation scales. Given the decay of community composition with distance (Socolar et al. 2016), it is likely that greater ecological equivalence would be achieved through a more local offset scheme.

Our analyses have some limitations. For example, price usually influences trade activity between buyers and sellers. Particularly for sellers, price is related to forgone opportunity costs but that is not the case for buyers, who expect price to be much lower than sellers’ forgone opportunity costs (Giannichi et al. 2018). Perhaps at smaller scales, where opportunity costs are high, there would be even less averted loss and more restoration because sellers would expect high returns of their surplus, making restoration a less costly offset option for buyers. However, this may not lead to any substantial impact on the overall additionality. We did not include price in this analysis because data specific to properties are inexistent. Some previous researchers accounted for price by using opportunity costs as a proxy (Bernasconi et al. 2016; Soares‐Filho et al. 2016) at the scale of municipalities, but we believe this reflects only sellers’ price preferences. More empirical data on price expectations would be useful in future analyses. Our algorithm was based on the assumption that all sellers were available for trade and that each buyer can find the optimal seller, which may not be true. However, we believe it would be arbitrary and unrealistic to establish criteria that would exclude nonparticipant sellers because there are no data that support this decision.

Our findings showed that limiting offsets to a small‐scale approach yielded greater additionality in a countrywide offset scheme. Although these findings were particular to the FC offset scheme, we believe that other offset schemes with similar options of restoration or buying credits elsewhere could benefit from the results of this study. When buyers are randomly or uniformly distributed in space, total additionality is likely to be less dependent upon the scale of the offset scheme. However, in most cases buyers’ distribution was aggregated in areas that are highly suitable for intensive agriculture (e.g., Fig. 1). Such areas are less likely to include cheap alternatives to restoration, but any offsetting area is likely to contribute to additionality (due to the overall suitability of the area for agriculture and the proximity of the area to infrastructure). As the allowed scale for offsetting increases, so does the market size because it moves into areas less suitable for agriculture (Santos et al. 2014). This phenomenon increases the chances of landowners finding cheap alternatives to restoration through credit buying. However, the areas acquired are far from infrastructure or are less suitable for agriculture, and as such they are at leaser risk of being developed (i.e., contribute less to additionality). Keeping restoration near the affected site could be particularly helpful for offset schemes in regions where development is predominant and restoration is feasible. Allowing offset schemes to occur more locally may be a way to incentivize the recovery of the lost habitat and avoid further loss in the surrounding landscape matrix. Given the vast amounts of degraded land and the recent global efforts to restore degraded landscapes (Verdone & Seidl 2017), localized strategies to promote regrowth may be a way to achieve ambitious restoration targets. The protection of natural vegetation remnants is important, but alone is not sufficient to deliver long‐term conservation goals (Chazdon & Guariguata 2016). Other large‐scale conservation strategies could also benefit from a think‐local focus to improve schemes’ additionality and maximize gains from averted loss and restoration.

## Supporting information

Compensation options and CRA (Appendix S1), land‐tenure data preparation (S2), land‐cover data (S3), classification of buyers and sellers (S4), matching algorithm (S5), and sensitivity analysis (S6) are available online. The authors are solely responsible for the content and functionality of these materials. Queries (other than absence of the material) should be directed to the corresponding author.
