# Supplementary material for: Scale dependency of conservation outcomes in a forest‐offsetting scheme
Source: Conserv Biol. 2019 Jul 30;34(1):148–57. doi: 10.1111/cobi.13362 (PMC7028087; doi:10.1111/cobi.13362)
Supplement: Supplementary file 2 [file COBI-34-148-s002.docx]

**Online Supporting Information**

**S1: Forest Code offset options**

The Brazilian Forest Code (FC) is the environmental legislation that controls and regulates native vegetation across the national territory within private properties. The FC states that every private property should set-aside areas of native vegetation with the purpose of conservation. The first type are the permanent protected areas, such as riparian and hilltop areas. These areas are protected by law and are not subject to legal deforestation. The second type, called Legal Reserves (LR), are areas of native vegetation that should be also maintained. The extent of Legal Reserves varies according to biome where the property is located. In the Amazon, for example, landowners should keep at least 80% of the property as standing native vegetation (see section S4 for more details). Landowners who have over cleared their LR until 2008^[[1]](#footnote-1)^ and have less than 80% of LR (named buyers) must offset their native vegetation deficit to be in compliance with the law. For that, the FC makes available offset options, which are described below.

*Offset in private lands inside Protected Areas (PAs)*

Some PAs in Brazil have been demarcated and established in areas where some private land was already in place. These private lands that are located inside PAs should be expropriated by the statutory environmental agency that must indemnify the owners so they can leave their land. In an attempt to alleviate the resources spent in this process, the FC allows buyers to offset their LR deficit by acquiring a private land inside PAs and donating to the environmental agency. In this case, buyers should by an entire private land that is at least equivalent to their LR deficit, including its standing native vegetation at whichever state included cleared areas (Brasil 2016). The price of such private lands inside PAs are determined by the property owners (sellers).

This strategy has been seen as problematic due to low additionality (as the offset occurs in areas that are already classified as protected, as they are inside a PA) and to over-surplus (Soares-Filho et al. 2016). Others consider positive as this could be an important resource to under-capitalized environmental agencies (Andrade, J., May, P.H. & Bernasconi 2013). Currently, properties inside PAs need to be authorized to be sold as a compensatory strategy. In 2017, there were seven properties habilitated to compensation, summing 295,975 hectares but it is likely that this number will increase as compliance starts to be adopted (Saretta, 2017).

*Environmental Reserve Quota (CRA, Portuguese acronym)*

Different from the offset inside PAs, CRA is a hectare-by-hectare offset option. Buyers who deforested their LR until 2008 may offset their deficit in other private properties that kept their LR above the minimum (> 80%, named sellers). Sellers issue quotas (1 hectare = 1 quota) and their acquisition is lease-based, i.e. buyers need to provide payments for sellers that, under a contract, will maintain their LR surplus as standing native vegetation. The FC does not establish minimal/maximum contract durations for CRA, so durations are agreed between buyers and sellers. As CRA is a lease, the native vegetation still belongs to the sellers. Trade must happen within the same biome and the same state. Cross-state trade is allowed if sellers are located inside priority conservation areas (figure S1). These conservation priority areas are not demarcated areas with established boundaries, they are simply areas of ecological interest or biodiversity hotspots. Considering that sellers have the right to legally deforest their surplus, CRA has the potential avoid future forest loss but still awaits regulation.

*Restoration*

Lastly, buyers have the option of on-site compensation. They can actively reforest their deficit or allow natural regrowth via abandonment.


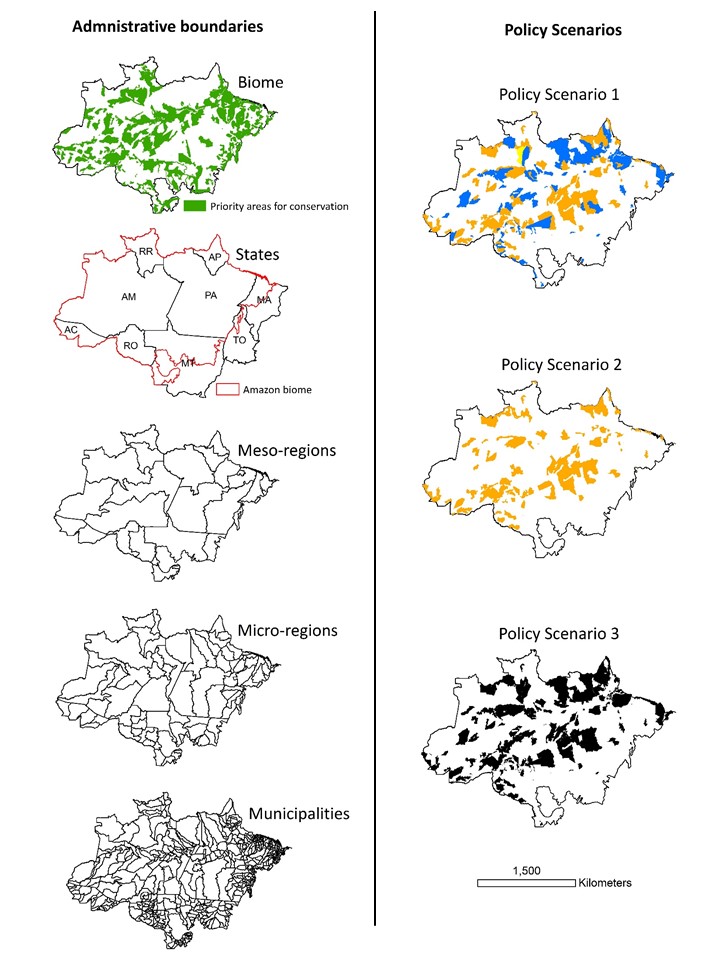


**Figure S1**. Left-hand column represents administrative boundaries used as the five spatial scales in which we simulated off-site compensation, from the largest to the smallest. The Amazon biome covers nine states in Brazil: Acre (AC), Amapá (AP), Amazonas (AM), Maranhão (MA), Mato Grosso (MT), Rondônia (RO), Roraima (RR) and Tocantins (TO). Maranhão, Mato Grosso and Tocantins are only partly covered by the Amazon, and therefore only properties located in the Amazon biome were considered in these three specific states. Areas covered in green at biome level represent priority areas for conservation, which were only used to simulate compensation in the entire biome (between states). Right-hand column represents the policy scenarios considered for each administrative boundary. Policy scenario 1 considered properties inside all PAs (federal – orange, state – blue, and municipal – yellow), scenario 2 only properties inside federal PAs, and scenario 3 does not account for PAs at all (black).

**S2: Land tenure data preparation**

Amazon land tenure was derived from Freitas et al. (2017), which is a database that integrates georeferenced land use categories including both private and public land. This land tenure map adopts a spatial resolution of 50m and contains the different categories of land use for all types of rural properties in Brazil. To meet the aims of this study, we first selected properties within the Amazon biome. Then, we excluded from this analysis polygons classified as non-processed land, as they encompass water bodies, roads and urban areas. Because participation in CRA is dependent upon clear land titling, we excluded properties classified as non-designated land. Although they cover a large portion of the Amazon (~55Mha), their clear designation and regularization process is slow and unclear (Freitas et al. 2017; Azevedo-Ramos & Moutinho 2018). We have also eliminated one category called simulated land. This category covers areas that are not registered in any official database therefore the authors decided to estimate land in these geographical voids. Hence is also uncertain if these lands have land titling. Land tenure data is available both at shapefile and raster formats. Manipulation was done using R, version 3.4.1 (R Core Team 2017).

**S3: Land cover data**

*GFC data*

We used Global Forest Change datasets (Hansen et al. 2013) to have an updated estimate of open land inside protected areas to explore potential regrowth. The most recent year available is 2016. Tree cover 2016 was calculated as tree cover in the year 2000 minus yearly loss until 2016, plus forest gain. As we were interested in open area, pixels representing forest loss from 2000-2016 were reclassified as open land. To match the Amazon land tenure map, forest/non-forest pixel size were resampled to 50m resolution and overlaid with the tenure map to calculate open area per property. We also used GFC forest cover of 2000 to apply in our classification of buyers and sellers (see next section). GFC data was processed in Google Earth Engine and exported as raster files to be further manipulated in ArcMap 10.3.1. We employed a threshold of 10% for canopy tree cover – the same established by United Nations Food and Agricultural Organization (Hansen et al., 2010) to also account for Cerrado areas within Amazon.

**Table S1.** Summary of datasets used and their respective source.

| Layer | Source |
| --- | --- |
| Rural Properties | Tenure Map of Brazil (Freitas et al. 2017) |
| Land cover | TerraClass 2008, 2014 (Almeida et al. 2016) |
| Forest and non-forest cover inside protected areas | Global Forest Change 2000-2016 (Hansen et al. 2013) |
| Permanent Protected Areas (hilltop and riparian areas) | CSR, UFMG (Soares-Filho et al. 2014) |
| Economic-ecological zoning and Priority areas for conservation | MMA^[[2]](#footnote-2)^ |

*TerraClass*

TerraClass is a project of the Brazilian Space Research Agency that maps land use and land cover changes across the Brazilian Amazon. TerraClass explicitly accounts for classes, such as secondary forest and regenerating pasture (which can both be accounted as native vegetation remnants) hence our preference for this dataset. TerraClass has 15 different land cover classes (see Almeida *et al.*, 2016). Our analysis does not require that level of detail so we reclassified the dataset, which resulted in: (1) Forest, (2) Secondary Forest, (3) Savannah, (4) Pasture, (5) Crop, and (6) Others. The main classes of deforested land were reclassified into pasture, crop and others. The classes corresponding to native vegetation were preserved as their original classification: forest, secondary forest and savannah. TerraClass has one specific class called “regenerating pasture”, which contains vegetation at successional stage. This category was reclassified into Secondary Forest. We resampled pixels to 50m resolution and then calculated for each property the area covered by each land cover class in ArcMap 10.3.1. We used the years 2008 and 2014 for this analysis. The FC states that only landowners who deforest until 2008 are eligible for off-site compensation. Thus the vegetation cover in 2008 was necessary to classify eligible buyers and 2014, eligible sellers.

*Hilltop and riparian areas*

Riparian vegetation and hilltop areas, if covered by natural vegetation, cannot be legally deforested. Riparian areas along rivers and streams have hierarchical levels of protection: the wider the river or stream, the larger the buffer of riparian protection (Brasil 2012). To have a precise estimate of the native vegetation occurring in these areas, we used the raster map provided by Soares-Filho *et al.* (2014), which contains the hierarchical buffers. Then, we calculated for each property the area corresponding to riparian buffers to later discount from our estimates of avoided deforestation (see main text) as these areas are not subject to legal deforestation. For the calculation of the total native vegetation per property these areas were included as the Forest Code allows the computation of riparian areas as part of LR and can be used as forest surplus in CRA. Hilltop areas also follow a hierarchical protection, but that is related to the slope and elevation (Brasil 2012). We applied the same procedure of the riparian areas here, also using the raster map by Soares-Filho et al. (2014). Hilltop areas that overlap with native vegetation were computed as Legal Reserve for the same reason as riparian areas and also discounted from the estimate of avoided deforestation.

At the end of this stage, our database contained private properties outside PAs with their respective natural vegetation remnants in 2008 and 2014; and private properties inside PAs with their respective amount of open land in 2016.

Importantly, to calculate potential avoided deforestation in our analysis, we discounted riparian and hilltop areas, as they are protected by law and cannot be deforested.

*Economic-Ecological Zoning and Priority Areas for Conservation*

The Ministry of Environment (MMA) establishes a zoning system with areas of specific designation, called which The Economic-Ecological Zoning (ZEE). There are three main zones: (1) agricultural expansion; (2) controlled uses; and (3) special uses. In areas designated to agricultural expansion, Legal Reserve is reduced to 50% in the Amazon (instead of 80%). Priority areas for conservation are areas with strong conservation recommendations and supposedly conservation actions. The implication of these areas to CRA is that at biome level, only sellers located in priority areas can offer their Legal Reserve surplus.

**S4: Classification of buyers and sellers**

To classify the properties into buyers and sellers is not a trivial task. The Forest Code states many exceptions to the rules, which makes it complex. To facilitate understanding, we depict this process in figure S1, but below we provide a detailed explanation.


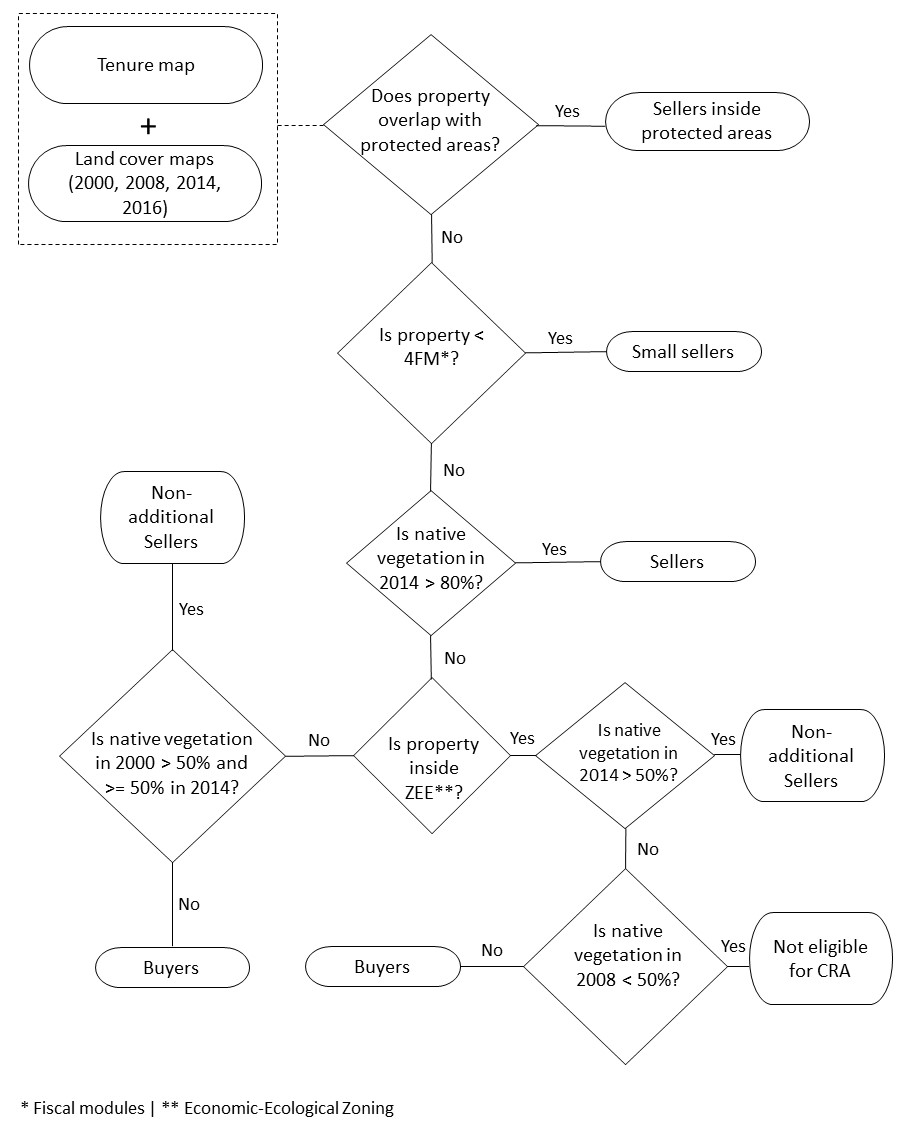
*Eligible sellers*

**Figure S2**. Depiction of the decision tree used in the classification of buyers and sellers using the land tenure dataset and land covers datasets from 2000, 2008, 2014 and 2016 with the later just to calculate potential regrowth inside protected areas.

As a general rule, all properties in the Amazon must maintain a minimum of 80% as standing vegetation. If a property has above 80% as native vegetation today (using TerraClass 2014 as a proxy), this property is considered a seller. Now we have the exceptions. If a property is located in the agricultural expansion zones and has Legal Reserve above 50%, it is a seller. All small properties, those who are inferior to four fiscal modules (a standard national unit that varies according to the municipality, between 50 and 100 in the Amazon) are also considered sellers and can offer whichever amount of native vegetation they have. Settlements are also considered sellers as they can offer their joint Legal Reserve for compensation. They are treated the same way as small landowners. Lastly, back in 2000, the Forest Code stated that the minimum Legal Reserve percentage was 50%. Article 68 says that landowners that supressed their Legal Reserve respecting the minimum percentage that was valid at that time and still maintain the same or a higher amount of native vegetation, can also offer their “surplus” in CRA market. To apply this exception we used the year 2000 of GFC dataset to calculate native vegetation for each property. Private properties that overlap with protected areas were also classified as sellers. In this case, the entire property is accounted as surplus. Thus, total surplus is a sum of all native vegetation above 80% or 50%, native vegetation in smallholdings and settlements, and private land inside protected areas.

*Eligible buyers*

We used TerraClass 2008 to classify properties eligible for CRA compensation. Only landowners who illegally deforested their native vegetation below the 80% minimum until 2008 are allowed to participate in CRA. In general, all landowners who have below 80% of native vegetation in 2008 are considered buyers. If the property is located in the agricultural expansion zones and has Legal Reserve below 50%, the property is also classified as buyer.

**S5: The best-match algorithm**

*Rationale and buyers’ decision-making process*

We developed an algorithm (the best-match algorithm) to simulate trade between potential buyers and sellers. The best-match algorithm was written by Marta Lisli Giannichi and Yoni Gavish, in R language (R Core Team, 2017) using the package foreach ({Microsoft} and Steve Weston 2017). The algorithm is deterministic since it finds the optimal deal for each buyer, and the order of deals is decided by the ‘level of optimality’ (the smallest difference between the buyer’s deficit and seller’s surplus). The algorithm is available in section S7 of this Supplementary Information and the data is available upon request.

We established two different phases for the algorithm to run and simulate offset between buyers and sellers. First, we attempted to exhaust buyers’ deficit with private lands inside PAs, by setting a parameter in the best-match as TRUE (sell.cu = T, see code below). Then, for the buyers who were not successful in finding a best-match to their deficit, we simulated CRA trade. Lastly, if both previous offset attempts were still unsuccessful, the buyer was automatically allocated to regrowth. This orderly fashion of decision-making from the buyers was guided based on a study conducted by Giannichi et al. (2018) that shows that buyers have strong preference for perpetual contracts. Given that private land inside protected allows for such perpetuity, we assumed that buyers would prefer this option. Thus, in policy scenarios 1 and 2, we ranked this option as buyers’ first choice. CRA as buyers’ second option was made based on the assumption that buyers would seek for another option of off-site offset strategy instead of forgoing productive land; and regrowth as their third option as this requires forgoing hectares of productive land. Buyers who succeeded in finding private land inside PAs to offset were considered compliant and, therefore, were not included in the subsequent runs. Buyers who did not find private land inside PAs were then included in the subsequent runs of the best-match algorithm to offset with CRA. If this was not successful and the buyer still had not found an area to offset, they were allocated to regrowth, as this as the third and last offset option.

*Trade inside PAs*

For offsets in PAs, buyers must purchase an area at least equivalent to their deficit. In order to not constrain the model to areas that were exactly the same size as the deficit, we assumed that buyers would be willing to purchase an area up to 20% larger than their deficit. Thus, there was a range of possible sellers inside PAs for a given buyer. For trade inside PAs we established only one transaction as buyers must purchase an entire property. This assumption likely reflects what a buyer would do. However, for the sellers, they might try to maximize their area and sell it to multiple buyers but this would require the property to be dismembered. For example, if a seller owns a property of 10,000 ha, they could potentially dismember it into 10 parts of 1,000 ha or two parts of 5,000 ha. Currently, there is currently no data or study that could provide guidance to include such parameter in the model.

The successful trade was made based on the smallest difference between the buyer’s deficit and the seller’s area. For example, if a buyer with 100 ha deficit found two sellers, one with 110 and another with 120 ha of area, the best-match was with the seller holding 110 ha. This buyer and the corresponding matching seller were then unavailable for other future trades. On the other hand, buyers who did not find a matching seller were available for the subsequent simulation (CRA trade, see below). This was iterated for each of the five administrative boundaries. We performed a sensitivity analysis (S6) to test whether our results were affected by the 20% limit established for successful trades by increasing such limit up to 150%.

*CRA trade*

CRA is a more flexible offset option. Because it is a hectare-by-hectare market, buyers can offset with multiple sellers and sellers can supply multiple buyers. In our model we allowed buyers and sellers to engage in three transactions. As stated in the main text, the transaction costs involved in CRA could be potentially high, as each contract needs to be registered in the notary office (Soares-Filho et al. 2016). Therefore, we assumed that both buyers and sellers would try to reduce transaction costs by maximizing the utility of their surplus/deficit with the least number of contracts possible.

In the first round, buyers select the most optimal deal with sellers. After finding the optimal deal, their respective deficits and surpluses are updated. For example, if a buyer who holds 100 ha of deficit found their best-match with a seller who holds 123 ha of surplus, the buyer becomes compliant and this seller still has 23 ha available for another buyer in the following round. Another possibility is if a buyer who holds 100 ha of deficit found his best-match with a seller who holds 80 ha of surplus. Then, the buyer is not compliant, as they still have 20 ha to offset, but the seller is not available anymore for the subsequent rounds. Each round, buyers and sellers’ status are updated. As long as a seller had more than 1 ha of surplus, they were available for trade. In the end of the simulation, buyers who did not manage to offset their deficit with three contracts had their remaining deficit allocated to regrowth.

Buyers and sellers with deficit and surplus smaller than 1 hectare were excluded from the protected areas and CRA trade. We assumed these landholders will not opt for neither of the compensation possibilities given the high transaction costs associated with them (May et al. 2015; Soares-Filho et al. 2016), just to offset a very small area.

*Step-by-step algorithm guide*

1. Identify the buyers (with contracts and deficit);
2. Identify the sellers (with contracts and surplus),
3. If there is at least 1 buyer and 1 seller, loop (foreach) over all buyers and for each one:
       3.1. get the buyer deficit
       3.2. To first exhaust trade inside PAs, set sell.cu = TRUE to subset all the sellers inside PAs that are within the 1.2 range of the buyer’s deficit (see code provided as a separate R file)
       3.3. If restricted by region, subset all the sellers in the region
       3.4. Calculate the delta of the focal buyer against all relevant sellers
       3.5.  Subset the seller that has the lowest delta from the focal buyer
       3.6.  Return the buyer-seller pair of 3.5
4. Bind the best buyer-seller pair returned in 3.6 for all buyers into a single data frame (T1, see code provided as a separate R file)
5. Get the single buyer-seller pair with the lowest delta (or Na otherwise).

**S6: Sensitivity analysis**

To know whether changes in our assumptions would substantially change the patterns found in our results, we submitted our simulations to a sensitivity analysis. Firstly, we tested if changing the buyers’ criteria of offsetting in private land inside protected areas that were up to 1.2 the size of their deficit would influence the amount of offset and, consequently, on CRA market. We ran the matching algorithm for compensation in both federal and state protected areas (policy scenario 1) at biome level. Besides the 1.2 limit, we tested five other limits (table S2).

The only limit that showed a substantial change in the total offset available for CRA was one. This means that buyers were looking for sellers inside protected areas that matched exactly their deficit. Other tested limits did not show a substantial impact on the amount of offset from total deficit. From 1.1 to 2.5 there was only 4% difference, on average, in total offset across each administrative boundary. For that reason, we decided to maintain our limit of 1.2 in simulations inside protected areas.

**Table S2.** Extent of offset and regrowth in private lands inside protected areas under different tolerance limits equal or above deficit size. For example, limit of 1 means area is equal to deficit; limit of 2 means the area is twice as large as the deficit. The percentage column indicates the percentages of offset inside protected areas from forest deficit.

| Administrative  boundary | Limit | Offset from total deficit (ha) | % from forest deficit | Regrowth (ha) |
| --- | --- | --- | --- | --- |
| Biome | 1 | 182621.5 | 3.6 | 21798.75 |
|  | 1.1 | 1293285 | 26.1 | 97246.5 |
|  | 1.2 | 1361218 | 27.5 | 100790.5 |
|  | 1.5 | 1462255 | 29.5 | 121265.5 |
|  | 2 | 1485974 | 30 | 121332 |
|  | 2.5 | 1497175 | 30.2 | 121445.5 |
| State | 1 | 0 | 0 | 0 |
|  | 1.1 | 971628.9 | 19.6 | 88051.75 |
|  | 1.2 | 1018956 | 20.6 | 97427.5 |
|  | 1.5 | 1139265 | 23 | 107282.8 |
|  | 2 | 1168476 | 23.6 | 107992.8 |
|  | 2.5 | 1181521 | 23.8 | 112055.5 |
| Meso | 1 | 0 | 0 | 0 |
|  | 1.1 | 613907.2 | 12.4 | 60535.25 |
|  | 1.2 | 683844 | 13.8 | 69148.5 |
|  | 1.5 | 744025 | 15 | 79429.25 |
|  | 2 | 776258.6 | 15.6 | 81044.25 |
|  | 2.5 | 797744.4 | 16.1 | 87708.75 |
| Micro | 1 | 0 | 0 | 0 |
|  | 1.1 | 392533 | 7.9 | 44031.75 |
|  | 1.2 | 477018.7 | 9.6 | 60189.25 |
|  | 1.5 | 532734.1 | 10.7 | 68165 |
|  | 2 | 583361.9 | 11.7 | 70536.75 |
|  | 2.5 | 600399 | 12.1 | 75856 |
| Muni | 1 | 0 | 0 | 0 |
|  | 1.1 | 205632.3 | 4.1 | 27513.25 |
|  | 1.2 | 248841.6 | 5 | 32701.25 |
|  | 1.5 | 312027.9 | 6.3 | 38770.75 |
|  | 2 | 350980.8 | 7.1 | 41576.25 |
|  | 2.5 | 366486.3 | 7.4 | 49332.25 |

We also tested if increasing the number of contracts for sellers would have an impact on regrowth or avoided deforestation. We simulated trade across the five administrative boundary allowing sellers to have up to 10 contracts and maintained the limit of 3 contracts for the sellers. We tested this limit under policy scenario 3 because there were no interference of regrowth inside protected areas resulting in a precise idea of the extent of regrowth when increasing the contract numbers.

Increasing the number of contracts did not change the pattern observed when the limit in the number of contracts for sellers was three (figure S2). Avoided deforestation still decreased as administrative boundary became smaller and the opposite happened with regrowth. However, there were some very minor changes in the extent of regrowth and avoided deforestation at biome, state and municipality levels (table S3). Regrowth at biome level increased in 6 hectares, at state level decreased in 85,531 hectares and at municipality level 6,650. Avoided deforestation decreased only at state level, in 38,134 hectares

Because this 10-contract increase did not substantially change the patterns observed, we did not test in other scenarios. Nevertheless, we are aware that as the market systems takes place, intermediaries may influence the extent of regrowth and avoided deforestation. Some surpluses are considerably large and can offset the demand of several large buyers. As suggested by previous studies, the over-surplus might have negative impacts on avoided deforestation (May et al. 2015; Soares-Filho et al. 2016).

**Table S3.** Changes in regrowth and avoided deforestation across all administrative boundaries, after increasing to 10 the number of contracts for sellers and maintaining three for buyers. We considered only **Policy Scenario 3** for this comparison. To better understand changes, we also provide regrowth and avoided deforestation areas obtained from the three contracts limits for both buyers and sellers.

| Administrative boundaries | Regrowth (ha) | | | Avoided deforestation (ha) | | |
| --- | --- | --- | --- | --- | --- | --- |
|  | 3 contracts | 10 contracts | Change | 3  contracts | 10 contracts | Change |
| Biome | 433 | 439 | +6 | 1,646,512 | 1,646,512 | 0 |
| State | 215,898 | 130,367 | -85,531 | 1,480,401 | 1,442,266 | -38,134 |
| Meso-region | 323,538 | 323,538 | 0 | 1,201,593 | 1,201,593 | 0 |
| Micro-region | 685,852 | 685,592 | 0 | 1,068,577 | 1,068,577 | 0 |
| Municipality | 1,315,092 | 1,308,442 | -6,650 | 846,073 | 846,073 | 0 |


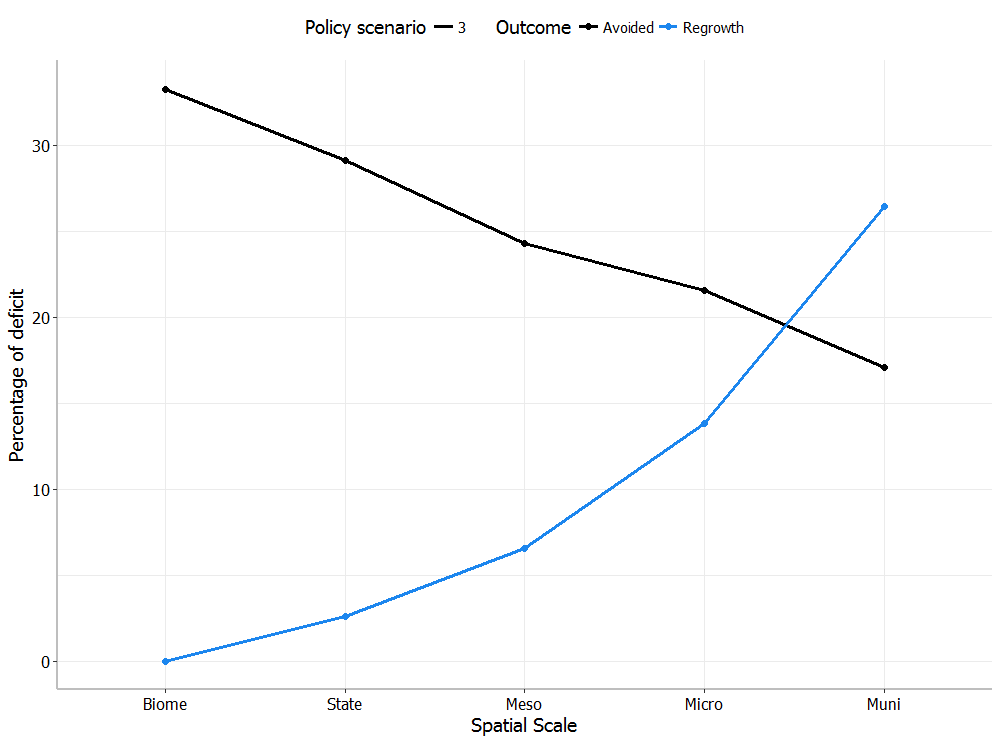


**Figure S3.** Patterns of avoided deforestation and regrowth after increasing sellers’ contracts to ten and keeping buyers’ at three, at the five different administrative boundaries. Only policy scenario 3 was used to test increase in contract number.

**S7: Best-match algorithm**

Please find R file with the code included as a separate file.

**References**

{Microsoft} and Steve Weston. 2017. foreach: Provides Foreach Looping Construct for R. Available from https://cran.r-project.org/package=foreach.

Almeida CA de, Coutinho AC, Esquerdo JCDM, Adami M, Venturieri A, Diniz CG, Dessay N, Durieux L, Gomes AR. 2016. High spatial resolution land use and land cover mapping of the Brazilian Legal Amazon in 2008 using Landsat-5/TM and MODIS data. Acta Amazonica **46**:291–302. Available from http://www.scielo.br/scielo.php?script=sci_arttext&pid=S0044-59672016000300291&lng=en&tlng=en.

Andrade, J., May, P.H. & Bernasconi P. 2013. A policy mixto finance protected areas in Mato Grosso, Brazil. Pages 379–394 in D. Springer, editor. Governing theprovision of ecosystem services.

Azevedo-Ramos C, Moutinho P. 2018. No man’s land in the Brazilian Amazon: Could undesignated public forests slow Amazon deforestation? Land Use Policy **73**:125–127. Elsevier. Available from https://doi.org/10.1016/j.landusepol.2018.01.005.

Brasil RF do. 2016. Issn 1677-7042 1. Diário Oficial da União **153**:30.

Brasil L 12651. 2012. Presidência da república.:3220–3304. Available from http://www.planalto.gov.br/ccivil_03/leis/L7802.htm.

Freitas FLM de, Sparovek G, Mörtberg U, Silveira S, Klug I, Berndes G. 2017. Offsetting legal deficits of native vegetation among Brazilian landholders: Effects on nature protection and socioeconomic development. Land Use Policy **68**:189–199.

Hansen MC et al. 2013. High-Resolution Global Maps of 21st-Century Forest Cover Change. Science **342**:850–853. Available from http://science.sciencemag.org/content/342/6160/850.abstract.

May PH, Bernasconi P, Wunder S, Lubowski R. 2015. Environmental reserve quotas in Brazil’s new forest legislation: an ex ante appraisal. CIFOR Occasional Paper:49.

R Core Team. 2017. R: A Language and Environment for Statistical Computing. Viena, Austria. Available from http://www.r-project.org/.

Saretta CB. 2017. Compensação de Reserva Legal em Unidade de Conservação: Trajetórias, Fortalezas, Oportunidades e Desafios. UNICAMP.

Soares-Filho B, Rajão R, Macedo M, Carneiro A, Costa W, Coe M, Rodrigues H, Alencar A. 2014. Cracking Brazil ’ s Forest Code. Science **344**:363–364.

Soares-Filho B, Rajâo R, Merry F, Rodrigues H, Davis J, Lima L, Macedo M, Coe M, Carneiro A, Santiago L. 2016. Brazil’s market for trading forest certificates. PLoS ONE **11**. Available from http://dx.doi.org/10.1371/journal.pone.0152311.

1. This cut-off date is established by the Forest Code. Buyers who deforested their LR after 2008 are not eligible for offsetting in PAs nor CRA and must restore their LR deficit. [↑](#footnote-ref-1)
2. <http://www.mma.gov.br/gestao-territorial/zoneamento-territorial/zee-nos-estados>. Last accessed: 21/02/2018 [↑](#footnote-ref-2)
